# Therapeutic Targeting of the Gas6/Axl Signaling Pathway in Cancer

**DOI:** 10.3390/ijms22189953

**Published:** 2021-09-15

**Authors:** Mai Tanaka, Dietmar W. Siemann

**Affiliations:** Department of Radiation Oncology, College of Medicine, University of Florida, Gainesville, FL 32610, USA; siemadw@ufl.edu

**Keywords:** receptor tyrosine kinase inhibitors, Gas6/Axl pathway, cancer therapeutics, small molecule inhibitors, therapeutic resistance

## Abstract

Many signaling pathways are dysregulated in cancer cells and the host tumor microenvironment. Aberrant receptor tyrosine kinase (RTK) pathways promote cancer development, progression, and metastasis. Hence, numerous therapeutic interventions targeting RTKs have been actively pursued. Axl is an RTK that belongs to the Tyro3, Axl, MerTK (TAM) subfamily. Axl binds to a high affinity ligand growth arrest specific 6 (Gas6) that belongs to the vitamin K-dependent family of proteins. The Gas6/Axl signaling pathway has been implicated to promote progression, metastasis, immune evasion, and therapeutic resistance in many cancer types. Therapeutic agents targeting Gas6 and Axl have been developed, and promising results have been observed in both preclinical and clinical settings when such agents are used alone or in combination therapy. This review examines the current state of therapeutics targeting the Gas6/Axl pathway in cancer and discusses Gas6- and Axl-targeting agents that have been evaluated preclinically and clinically.

## 1. Introduction

Receptor tyrosine kinases (RTKs) are cell surface receptors that mediate a number of physiological responses and homeostasis. However, gene amplification, overexpression, and activating mutations of RTKs are often associated with cancer development, progression, and metastasis and have served as pharmacological targets in cancer treatment. Axl belongs to the TAM (Tyro3, Axl, MerTK) subfamily of the RTKs. Physiologically, the Gas6/Axl pathway plays an important role in platelet aggregation and vessel integrity [1,2,3]. Axl knockout in germ cells does not result in embryonic lethality [4]. The growth arrest specific 6 (Gas6) protein belongs to the vitamin K-dependent family of proteins and is a high affinity ligand for Axl. Overexpression and activation of Axl are widely observed in various cancer types and have been implicated in multiple steps of cancer pathogenesis. In addition, high Axl expression and activation are associated with poor prognosis, outcome, and resistance to therapy in cancer patients [5,6,7,8,9,10,11]. As such, the Gas6/Axl pathway has gained attention as a promising therapeutic target for drug development in multiple tumor types. In this review, we present an overview of the Gas6/Axl signaling pathway and highlight the Gas6 and Axl inhibitors that are being investigated preclinically and clinically.

## 2. Gas6/Axl Pathway in Cancer

Axl was first isolated from chronic myelogenous leukemia cells in 1988 [12]. Structurally, Axl contains two immunoglobulin-like (IgL) domains, two fibronectin III (FNIII) domains, a transmembrane domain, and an intracellular kinase domain [13]. The Axl pathway can be activated by its ligand Gas6, which belongs to the vitamin K-dependent family of proteins. Gas6 contains the γ-carboxyglutamic acid (Gla) domain, four epidermal growth factor (EGF)-like domains, and two laminin G-like (LG) domains [14,15]. The N-terminus Gla domain mediates binding to phosphatidylserine expressed on cell membranes, apoptotic cells, and debris [16,17]. Gas6 and Axl interact via the first LG-like domain of Gas6 and the two IgL domains of the Axl ectodomain [18]. Gas6 and Axl bind at a one-to-one receptor-to-ligand ratio and then dimerize with another Gas6–Axl complex to initiate a downstream signaling cascade.

**Figure 1:**
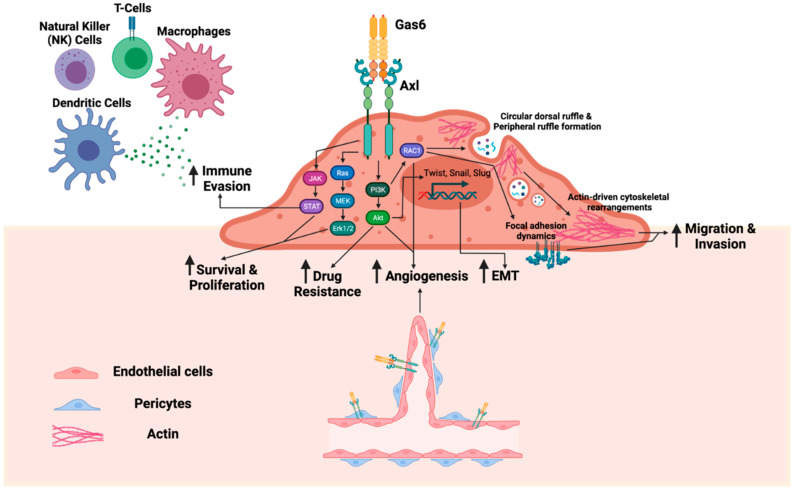
The Gas6/Axl pathway mediates multiple steps of the metastatic cascade. Upon Axl binding to its ligand growth arrest specific 6 (Gas6) protein, Axl dimerizes and autophosphorylates its tyrosine residues in the kinase domain. Axl activation regulates downstream signaling, including the JAK/STAT pathway, Ras/MEK/Erk1/2 pathway, and PI3K/Akt pathway. In turn, the Gas6/Axl pathway upregulates pro-tumorigenic functions, such as immune evasion, survival, proliferation, drug resistance, angiogenesis, epithelial-to-mesenchymal transition (EMT), migration, and invasion. Gas6 and Axl are also expressed by stromal cells, including endothelial cells, pericytes, and subsets of immune cells, to promote tumor progression and metastasis.

The role of Gas6 and Axl in cancer has been reviewed previously [19,20,21,22]. Briefly, Axl is overexpressed in many cancer types and is associated with therapeutic resistance, poor clinical prognosis, and worse outcome [23,24,25,26]. Axl also mediates key components of the metastatic cascade, including, but not limited to, epithelial-to-mesenchymal transition, migration and invasion, proliferation, survival, stemness, angiogenesis, and immune evasion [19,20,22,27] (Figure 1). A study by Zdzalik-Bielecka and colleagues demonstrated that the Gas6/Axl pathway mediates actin cytoskeleton remodeling for cell migration and invasion via formations of peripheral ruffles and circular dorsal ruffles [28]. Such Gas6-induced Axl activation contributes to focal adhesion turnover, cell spreading, and elongation through the activation of PI3K and RAC1 [28,29]. Another study similarly demonstrated that Axl mediates cell invasion through the regulation of lysosome peripheral distribution [30].

In the tumor immune microenvironment, Axl signaling in tumor cells polarizes tumor-associated macrophages toward the M2 phenotype via the Axl/Pi3K/Akt/NF-kB pathway [31]. Axl inhibition elicits a favorable immune response, characterized by a decreased infiltration of CD206^+^ macrophages and neutrophils and an increased infiltration of CD45^+^ immune cells, CD8^+^ T cells, CD4^+^ T cells, and I-A/I-E^+^ macrophages [32].

Additionally, it is becoming increasingly clear that the Gas6/Axl signaling axis also impacts non-neoplastic cell populations, which may be of particular relevance when viewed in the context of the tumor microenvironment. For example, pericyte FAK expression is a negative regulator of tumor angiogenesis and tumor growth [33]. In FAK-depleted pericytes, activated Pyk2 increases Gas6 transcript levels and signals via the Gas6/Axl/Akt/Cyr61 pathway to promote tumor cell proliferation and angiogenesis [34,35]. Axl is also expressed by bone marrow-derived cells, dendritic cells, monocytes, natural killer cells, and platelets [36,37,38,39,40,41,42]. A study by Tirado-Gonzalez and colleagues also demonstrated that Axl-expressing leukemia-associated macrophages contribute to immune suppression and impair the functions of NK- and T-cell-mediated tumor cell killing [43].

## 3. Therapeutic Targeting of Gas6

### 3.1. Warfarin

Warfarin is a vitamin K antagonist that inhibits the enzyme vitamin K epoxide reductase to recycle inactive vitamin K epoxide to its active form. Warfarin blocks vitamin K-dependent gamma-carboxylation of the γ-carboxyglutamic acid-rich (Gla) domain of Gas6 [44], which prevents the Gas6 interaction with externalized phosphatidylserine on the surface of apoptotic cells and debris (Table 1). Preclinically, the ability of warfarin to impair Gas6 function has been investigated in pancreatic cancer, lung cancer, melanoma, and breast cancer models [42,45,46,47]. Zweemer and colleagues demonstrated that warfarin disrupts the phosphatidyl serine (PS)–Gas6 interaction and consequently impairs tumor cell migration [47]. In addition, Paolino and colleagues demonstrated that warfarin inhibits Gas6-mediated activation of TAM receptors on NK cells and exerts anti-metastatic activity [42]. While an early Phase I study to evaluate the effect of warfarin on markers of the Axl pathway was initiated in 2019, the study was withdrawn in 2021 due to lack of accrual (Clinical Trial Identification #: NCT03536208).

### 3.2. Soluble Receptors

#### 3.2.1. MYD1/MYD1-72

MYD1 is an Axl decoy receptor with a high affinity for Gas6 (Table 1) [48]. MYD1 contains the major site of Axl Ig1 with four mutations to improve binding to Gas6 [48]. MYD1 inhibited Gas6-mediated Axl-, Akt-, and Erk-phosphorylation and decreased the number of peritoneal metastases in an ovarian cancer model [48].

MYD1-72 is a second-generation Axl decoy receptor that has a higher binding affinity than that of MYD1 (K_D_: 420 fM vs. 93 fM, respectively). In preclinical studies, MYD1-72 decreased Axl phosphorylation and impaired tumor growth and metastatic burden in vivo. A combination of MYD1-72 with gemcitabine significantly improved the survival of mice with pancreatic cancer to 57 days compared to 17 days in the control and MYD1-72 alone groups and 35 days in the gemcitabine group. A clinical evaluation of this compound has not been reported.

#### 3.2.2. AVB-500

AVB-500 is an Axl decoy receptor with an affinity of 150 fM for Gas6 (Table 1). AVB-500 has been preclinically investigated in renal cell carcinoma and ovarian cancer. The results showed that treatment with AVB-500 decreases Gas6-induced Axl and Src phosphorylation, tumor vessel density, tumor growth, and metastatic burden [49,50,51].

AVB-500 is undergoing Phase I/II clinical trials for patients with platinum-resistant or recurrent ovarian, fallopian tube, or peritoneal cancers as a combination therapy (Clinical Trial Identification #s: NCT03639246, NCT04019288). Three other studies are currently recruiting patients for ovarian cancer, renal cell carcinoma, and pancreatic adenocarcinoma to evaluate its efficacy as a combination therapy with paclitaxel, cabozantinib, and nab-paclitaxel/gemcitabine, respectively (Clinical Trial Identification #s: NCT04729608, NCT04300140, NCT04983407).

## 4. Therapeutic Targeting of Axl

### 4.1. Type I Kinase Inhibitors

Small molecular kinase inhibitors can be classified based on the structure of the protein–inhibitor complex [52]. The N-terminal Asp-Phe-Gly (DFG) motif of the activation loop is conserved and regulates kinase activity. Type I protein kinase inhibitors bind to the active protein kinase conformation or DFG-in conformation.

#### 4.1.1. Bemcentinib (BGB324, R428)

Bemcentinib is a highly specific and selective Axl inhibitor with an IC_50_ of 14 nM in cellular assays (Table 2) [53,54]. This agent has been widely studied in the laboratory in a variety of cancer models, including breast, prostate, lung, pancreatic, and ovarian cancer, and has been shown to decrease tumor cell migration, invasion, and colony formation in vitro and impair primary tumor growth, immune cell infiltration, and metastasis in vivo [55,56,57,58,59,60,61,62]. Axl inhibition alters the expression patterns of EMT markers, upregulating epithelial markers, such as E-cadherin, and downregulating mesenchymal markers, such as N-cadherin, ZEB1, Snail, Slug, Twist, and MMP9 [63,64,65]. Furthermore, several studies have demonstrated that Axl inhibition reverses the therapeutic resistance of certain chemotherapies. Axl has also been associated with immune evasion, and the systematic treatment of tumor-bearing mice with bemcentinib has led to a reduction in tumor-infiltrating host cells, most notably cells of the myeloid lineage [66].

Bemcentinib entered clinical trials as the first Axl-specific inhibitor and is undergoing Phase I/II clinical trials for melanoma, non-small-cell lung cancer (NSCLC), mesothelioma, acute myeloid leukemia, glioblastoma, and pancreatic adenocarcinoma (Clinical Trial Identification Numbers: NCT02872259; NCT02922777; NCT03184571; NCT03654833; NCT03824080; NCT03965494; NCT03649321). A Phase II clinical trial of bemcentinib in combination with pembrolizumab for patients with triple-negative breast cancer has been completed, but the results are not yet available (Clinical Trial Identification #: NCT03184558).

#### 4.1.2. Amuvatinib (MP-470)

Amuvatinib is a c-Kit/Axl kinase inhibitor (Table 2), with higher selectivity against c-Kit than against Axl (IC_50_ < 1 μM in cellular assays). Amuvatinib has been studied in models of gastrointestinal stromal tumors, lung cancer, and prostate cancer in the context of overcoming therapeutic resistance [60,67,68]. In the gastrointestinal stromal tumor (GIST) model, Mahadevan and colleagues showed that an imatinib-resistant GIST cell line overexpressed Axl compared to its parental cell line [68]. Treatment with amuvatinib showed synergistic, cytotoxic effects with docetaxel [68]. In an erlotinib-resistant lung cancer model, Axl inhibition by amuvatinib restored sensitivity to erlotinib [67].

Clinically, amuvatinib is undergoing Phase I/II trials for small-cell lung cancer (SCLC) and other unresectable or metastatic solid tumors (Clinical Trial Identification Numbers: NCT01357395; NCT00894894; NCT00881166). In a Phase I, dose-escalation study of patients with advanced solid tumors, amuvatinib was well tolerated up to 1500 mg/day with no report of dose-limiting toxicities; indeed, the study did not reach the maximum tolerated dose [69]. In a Phase Ib study of amuvatinib in combination with standard of care therapies for adults with advanced solid tumors, 12% demonstrated partial response to amuvatinib combined with paclitaxel/carboplatin or carboplatin/etoposide in neuroendocrine, NSCLC, and SCLC tumors [70].

#### 4.1.3. Dubermatinib (TP-0903)

Dubermatinib is an Axl inhibitor that has additional activity against Aurora-A and -B and Janus kinase 2 (JAK2). Dubermatinib has been studied in breast cancer, acute myeloid leukemia, neuroblastoma, and chronic lymphocytic leukemia models (Table 2) [71,72,73,74]. Dubermatinib impairs tumor cell migration, invasion, survival, proliferation, and colony formation and can affect chemotherapeutic agent sensitivity [75,76,77,78]. At the molecular level, it inhibits Axl signaling and Aurora B activation to induce G2/M cell cycle arrest [77]. In CLL B cells, dubermatinib induced apoptosis through the downregulation of Mcl-1, Bcl-2, and XIAP and the upregulation of BIM [72]. In a neuroblastoma model, a phospho-kinase array of dubermatinib-treated cells showed greater than 50% increases in p53 S^392^ and Chk-2 T^68^ phosphorylation, in line with a corresponding increase in histone H2A.X phosphorylation [73].

Dubermatinib has been studied as monotherapy and combination therapy. Preclinically, combination strategies with all-trans retinoic acid (ATRA), cisplatin, and VP16 demonstrated synergistic, cytotoxic effects [73]. Aveic and colleagues showed that a dubermatinib/ATRA combination in neuroblastoma cell lines was particularly effective against CD133^+^ cells, a marker associated with chemo- and radio-resistance [79,80,81]. Phase I/II studies are ongoing for patients with advanced metastatic or progressive solid tumors and AML to determine the dose, safety, pharmacokinetics, and pharmacodynamics of dubermatinib (Clinical Trial Identification Numbers: NCT02729298, NCT04518345, NCT03013998). A Phase I/II clinical trial investigating dubermatinib monotherapy and combination therapy with ibrutinib for patients with chronic lymphocytic leukemia was terminated (Clinical Trial Identification #: NCT03572634).

#### 4.1.4. Crizotinib (XALKORI^®^, PF-02341066)

Crizotinib is a multitargeted, small molecule tyrosine kinase inhibitor that inhibits c-Met, ALK, Ron, and Axl (IC_50_-Axl = 294 nM in cell-free assay) (Table 2). Crizotinib has been studied in a variety of cancer types, including NSCLC, gastric carcinoma, glioblastoma, anaplastic large-cell lymphoma, and leiomyosarcoma [82,83,84,85]. With respect to the role of crizotinib on the Axl signaling pathway, Dantas-Barbosa and colleagues showed that crizotinib impaired Axl phosphorylation, cell growth, cell cycle, and colony formation in leiomyosarcoma cells [82].

Crizotinib has been approved by the FDA to treat patients with ALK- or ROS1-positive NSCLC and pediatric patients with relapsed or refractory ALK-positive systemic anaplastic large-cell lymphoma (ALCL) [86,87]. There are 151 registered studies associated with crizotinib in the ClinicalTrials.gov database, and 44.4% (67 studies) are currently recruiting, enrolling by invitation, or active/not recruiting for monotherapy and combination therapy strategies. Acquisition of crizotinib resistance is common [88,89], typically developing within a few years for anaplastic lymphoma kinase-rearranged NSCLC [88]. Crizotinib-resistant NSCLC cells showed overexpression of Axl, epithelial-to-mesenchymal transition, and the acquisition of cancer stem cell-like properties [88,90,91].

#### 4.1.5. Bosutinib (BOSULIF^®^, SKI-606)

Bosutinib is a second-generation, multitargeted, small molecule tyrosine kinase inhibitor that targets Src, Abl, TGFβ, and Axl (Table 2). Bosutinib has been studied in BCR-ABL-dependent diseases, breast cancer, colorectal cancer, and NSCLC [92]. Bosutinib decreases Gas6-mediated Axl phosphorylation, migration, and invasion [93,94,95]. An immunoblot of bosutinib-treated cells showed decreased phosphorylation of Src pY^419^, FAK Y^576/577/925^, Pyk2 Y^580^, and p130Cas Y^410^ [93]; expression of vimentin and Slug; and increased expression of E-cadherin [96]. In a preclinical thyroid cancer model using Thrb^PV/P^Pten^+/-^ mice, bosutinib treatment significantly prolonged survival and impaired the development of lung metastases [96].

Bosutinib was approved by the FDA to treat patients with chronic, accelerated, or blast-phase Philadelphia chromosome-positive (Ph^+^) chronic myelogenous leukemia [97]. Bosutinib monotherapy and combination therapy with chemotherapy and other small molecule inhibitors are also being investigated in patients with breast cancer, glioblastoma, and other advanced solid cancers (Clinical Trial identification #s: NCT00319254, NCT01331291, NCT01001936).

#### 4.1.6. S49076

S49076 is a potent inhibitor of c-Met, as well as Axl, MerTK, and FGFR (Table 2). S49076 has been studied preclinically in gastric, lung, and liver cancer models [98,99] and has been shown to decrease the phosphorylation of Axl in a dose-dependent manner. S49076 impairs tumor cell viability, migration, and colony formation in vitro and tumor growth in vivo [98]. Clémenson and colleagues demonstrated that the mechanism of action of S49076 is dependent upon c-Met signaling dependency [100,101,102], where S49076 targets Aurora B kinase in c-Met-independent cells [99].

In a Phase I open-label study in patients with advanced solid tumors, a recommended once-daily dose of 600 mg was defined [103]. While 81.4% of patients had drug-related adverse events, the majority of the adverse events were grade I–II [103]. In a Phase I/II study in combination with bevacizumab in patients with recurrent glioblastoma, the combination did not improve patient survival outcomes [104]. The recommended Phase II dose was confirmed at 600 mg once daily, and no additional dose-limiting toxicity was observed [105,106].

#### 4.1.7. Sunitinib (SU111248)

Sunitinib is an oral, multitargeted, small molecule receptor tyrosine kinase inhibitor that targets VEGFR2, PDGFRβ, c-Kit, and Axl (Table 2). Sunitinib is approved by the FDA to treat patients with gastrointestinal stromal cancer, advanced RCC, and progressive, well-differentiated pancreatic neuroendocrine tumors [107,108]. However, Axl overexpression is associated with sunitinib resistance in renal cell carcinoma cells [109,110]. In patients with renal cell carcinoma, elevated Axl expression is associated with shorter overall survival [109]. Genetic and pharmacologic Axl inhibition in sunitinib-resistant cell lines demonstrate decreased tumor cell migration, invasion, EMT, and angiogenesis [109].

#### 4.1.8. SNS-314

SNS-314 is an aurora kinase inhibitor that also targets Axl, with an IC_50_ of 84 nM in a cellular assay (Table 2). SNS-314 impairs colony formation, histone H3 phosphorylation, and tumor growth in vivo [111,112]. In 2007, SNS-314 entered a Phase I study for patients with advanced solid tumors to evaluate safety and tolerability (Clinical Trial Identification Number: NCT00519662). The maximum tolerated dose was not established, and no responses were observed [113].

#### 4.1.9. Other Type I Axl Kinase Inhibitors Undergoing Preclinical Evaluation

NA80x1 is a 3-quinolinecarbonitrile that inhibits Axl phosphorylation, tumor cell migration, and invasion [94]. The IC_50_ of NA80x1 against Axl is 4.11 μM in a cellular assay and demonstrates inhibitory activity against Src kinase [94,114]. SGI-7079 is a selective Axl inhibitor that reduces Gas6-induced Axl phosphorylation and impairs the growth of mesenchymal NSCLC xenograft tumors [115]. In addition, Axl inhibitor DP-3975 [116,117], a TAM kinase inhibitor UNC569 [118], and MerTK/Flt3 dual inhibitor UNC2025 [119] are also type I Axl inhibitors with promising preclinical results.

### 4.2. Type II Kinase Inhibitors

Unlike type I protein kinase inhibitors, type II protein kinase inhibitors prefer to bind the inactive, DFG-out conformation.

#### 4.2.1. Cabozantinib (BMS-907351)

Cabozantinib is an oral, multitargeted, small molecule tyrosine kinase inhibitor that targets VEGFR, c-Met, and Axl (Table 3). Cabozantinib has been studied in RCC, esophageal squamous cell carcinoma, and lung cancer. Preclinically, cabozantinib treatment decreased Axl phosphorylation and TGF-β-induced E-cadherin expression, cell viability, migration, and tumor growth [120,121].

Cabozantinib has been approved by the FDA for patients with advanced renal cell carcinoma and sorafenib-resistant hepatocellular carcinoma. In January 2021, combination therapy of cabozantinib with nivolumab was approved as first-line treatment for patients with advanced renal cell carcinoma. The efficacy of the combination therapy versus sunitinib alone was evaluated in CHECKMATE-9ER, a randomized, open-label trial in patients with previously untreated advanced renal cell carcinoma [122] (Clinical Trial Identification: NCT03141177). The trial demonstrated significant improvement in progression-free survival, overall survival, and confirmed overall response rate for patients in the combination therapy arm compared with those who received sunitinib monotherapy [122].

#### 4.2.2. Foretinib (GSK1363089)

Foretinib is an oral, small molecule kinase inhibitor of c-Met, VEGFR, and Axl. Foretinib has been studied in esophageal squamous cell carcinoma, breast cancer, leiomyosarcoma, head and neck cancer, and gastric cancer (Table 3) [11,82,123,124,125]. Foretinib decreases Axl and Akt phosphorylation and impairs tumor cell viability and xenograft tumor growth [11]. In a lapatinib-resistant, HER2-positive, ER-positive breast cancer model, the foretinib-mediated downregulation of Axl restored lapatinib sensitivity [126].

Clinically, foretinib has been studied as a monotherapy and a combination therapy. In a Phase I dose-escalation study in patients with advanced solid tumors, the recommended maximum tolerated dose of foretinib was identified [127] (Clinical Trial Identification #: NCT00742131). Stable disease and partial responses were identified in 22 and 3 cases, respectively [127]. In a Phase I study of foretinib in combination with lapatinib in patients with HER2-positive metastatic breast cancer, no responses or improvement in progression-free survival were observed [123].

#### 4.2.3. Sitravatinib (MGCD516)

Sitravatinib is a multitargeted, small molecule receptor tyrosine kinase inhibitor that inhibits c-Kit, PDGFRα/β, c-Met, and Axl (Table 3). Sitravatinib has been shown to decrease Axl phosphorylation, colony formation, angiogenesis, and proliferation in sunitinib- or axitinib-resistant cells, as well as impairing tumor growth inhibition in vivo [128,129,130]. Du and colleagues demonstrated that sitravatinib alters the immune landscape of tumors by reducing the number of tumor-associated immunosuppressive myeloid cells and increasing the number of CD4^+^ T cells and proliferating CD8^+^ T cells [130]. Combination treatment of sitravatinib and PD-1 blockade demonstrated complete remission in 2 out of 14 mice with E0771 tumors [130].

In the clinic, sitravatinib is being investigated for use as monotherapy and combination therapy. A Phase I study is currently ongoing for patients with advanced cancer to evaluate its safety, pharmacokinetic, metabolism, pharmacodynamic, and clinical activity profiles (Clinical Trial Identification #: NCT02219711). In addition, several Phase I/II studies are underway to evaluate sitravatinib in combination with immune checkpoint inhibitors (Clinical Trial Identification #s: NCT03015740, NCT04734262, NCT03666143, NCT02954991).

#### 4.2.4. Glesatinib (MGCD265)

Glesatinib is an oral, multitargeted tyrosine kinase inhibitor that targets c-Met, VEGFR, Ron, Tie-2, and Axl (Table 3). Glesatinib impairs tumor angiogenesis and upregulates the expression of PD-L1 [130]. Du and colleagues demonstrated that combination therapy of glesatinib with anti-PD1 therapy delayed tumor growth [130]. As such, a Phase II study of glesatinib in combination with nivolumab in patients with non-small-cell lung cancer is ongoing (Clinical Trial Identification #: NCT02954991).

#### 4.2.5. Rebastinib (DCC-2036)

Rebastinib is a Bcr-Abl inhibitor that also impairs Src family proteins, c-Met, Tie-2, and Axl (Table 3). In a preclinical study utilizing breast cancer models, rebastinib impaired Axl phosphorylation, colony formation, cell cycle distribution, migration, invasion, and tumor growth and pulmonary metastasis in vivo [131]. Phase I/II studies are ongoing for patients with advanced metastatic or progressive solid tumors, breast cancer, and chronic myeloid leukemia to evaluate its safety, pharmacokinetics, and pharmacodynamics as monotherapy (Clinical Trial Identification Number: NCT00827138) or combination therapy with paclitaxel (NCT03601897, NCT02824575), eribulin (NCT02824575), and carboplatin (NCT03717415).

#### 4.2.6. Merestinib (LY2801653)

Merestinib is an oral, multitarget kinase inhibitor that impairs c-Met, Axl, Ron, and Flt3 (Table 3) [132]. Merestinib impairs cell proliferation, angiogenesis, and tumor growth in vivo [132,133]. Merestinib is being investigated clinically in multiple indications, including advanced or metastatic solid tumor, biliary tract cancer, non-small-cell lung cancer, and chronic myeloid leukemia, as a monotherapy or combination therapy (Clinical Trial Identification #s: NCT02711553, NCT02920996, NCT03125239). A Phase I study of merestinib with or without cetuximab, cisplatin, or gemcitabine identified the recommended dose at 120 mg once daily [134] (Clinical Trial Identification #: NCT01285037). One complete response and three partial responses were observed in patients with cholangiosarcoma [134].

#### 4.2.7. BMS-777607

BMS-777607 is a small molecule kinase inhibitor designed against c-Met, but it also has activity against Axl, Ron, and Tyro3 (Table 3). BMS-777607 has been studied in multiple indications, including prostate cancer, breast cancer, brain cancer (glioma/GBM), and fibrosarcoma [135,136,137,138,139,140]. BMS-777607 inhibits HGF-induced Met-autophosphorylation, colony formation, migration, and invasion [137,138]. In vivo, BMS-777607 impaired KHT sarcoma pulmonary metastases [135]; however, this was not the case for DU-145 and MDA-MB-231-4175-LM2 models [140]. Phase I/II clinical trials have been completed for patients with advanced or metastatic solid tumors, but the results are not yet available (Clinical Trial Identification #s: NCT01721148, NCT00605618).

#### 4.2.8. RXDX-106

RXDX-106 is an oral, small molecule inhibitor targeting the TAM receptors and c-Met (Table 3). RXDX-106 has been characterized in colorectal and breast cancer models and has been shown to impair Axl, Tyro3, MerTK, Akt, and Erk phosphorylation; tumor growth in vivo; and infiltration of leukocytes, macrophages, NK cells, and CD8^+^ T cells [141]. While an early Phase I study to evaluate RXDX-106 safety, tolerability, pharmacokinetics, pharmacodynamics, and clinical activity was planned, the study was terminated in 2018 (Clinical Trial Identification #: NCT03454243).

#### 4.2.9. Other Type II Axl Kinase Inhibitors Undergoing Preclinical Evaluation

LDC1267 is a selective TAM kinase inhibitor that provides anti-metastatic potential by enhancing NK cell activity in vivo [42]. In addition, NPS-1034 is a c-Met/Axl inhibitor that is efficacious against EGFR-TKI resistant non-small-cell lung cancer cells with c-Met and Axl activation [142].

### 4.3. Monoclonal Antibodies

#### 4.3.1. 20G7-D9

20G7-D9 has been studied in pancreatic cancer and triple-negative breast cancer models (Table 1) [143,144]. In pancreatic cancer cell lines Panc-1 and BxPC-3, 20G7-D9 reduced Gas6-induced Axl and Akt phosphorylation and impaired tumor cell migration, viability, and tumor growth in vivo [143]. Similarly, in a triple-negative breast cancer model, 20G7-D9 decreased tumor growth in vivo and silenced the Gas6-induced upregulation of EMT markers, including Snail, Slug, Twist, and Zeb1/2 [144].

#### 4.3.2. Axl-107-MMAE

Axl-107-MMAE is an antibody drug conjugate, containing human Axl antibody linked to the microtubule-disrupting agent monomethyl auristatin E (Table 1) [145]. Axl-107-MMAE induces cytotoxicity in vitro and impairs tumor growth in cervical cancer, lung cancer, and melanoma models [145]. In addition, combination treatment with MAPK pathway inhibitors cooperatively inhibited melanoma patient-derived xenograft growth [145]. The clinical safety and efficacy of Axl-107-MMAE are currently being evaluated in a Phase I/II clinical study for patients with advanced solid tumors (Clinical Trial Identification #: NCT02988817).

#### 4.3.3. BA3011

BA3011 is an anti-Axl humanized monoclonal antibody conjugated to monomethyl auristatin E using a cleavable linker (CAB-Axl-ADC) (Table 1). BA3011 is capable of reversibly binding to recombinant Axl and Axl-expressing cells in the tumor microenvironment but not in normal tissues. Preclinical studies demonstrate that BA3011 induces cytotoxicity in vitro and impairs tumor growth in lung, prostate, and pancreatic xenograft models [146]. The clinical safety and efficacy of BA3011 are currently being evaluated in Phase I/II and II clinical studies for patients with solid tumors, NSCLC, and ovarian cancers (Clinical Trial Identification #s: NCT03425279, NCT04681131, NCT04918186).

#### 4.3.4. Other Monoclonal Axl Antibodies Undergoing Preclinical Evaluation

YW327.6S2 is a phage-derived monoclonal antibody that recognizes both human and murine Axl [147]. In NSCLC and breast cancer models, YW327.6S2 impaired xenograft tumor growth, reduced breast cancer metastasis, and enhanced the efficacy of erlotinib as well as chemotherapy when used in combination [147]. Mab173 has been demonstrated to induce Axl degradation and impair tumor cell migration, invasion, apoptosis, and xenograft tumor growth in a Kaposi sarcoma model [148].

### 4.4. Axl-Specific Chimeric Antigen Receptor (CAR) T Cells

T cells with a chimeric antigen receptor (CAR) consisting of a single-chain variable fragment against Axl have been developed and evaluated preclinically. In a triple-negative breast cancer model, Axl-CAR-T cells inhibited tumor growth and showed association with increased secretion of antitumor cytokines, including TNFα, IFNγ, IL-2, IL-6, and IL-17A (Table 1) [149,150]. Axl-CAR.C7R (Axl-targeting CAR-T cells) that co-express constitutively activated the IL-7 receptor (C7R), showed enhanced activation of Axl-CAR-T cells in vitro, and impaired xenograft tumor growth (Table 1) [151].

In the clinical landscape, CCT301 CAR-T is undergoing a Phase I/II study for patients with renal cell carcinoma to assess safety, tolerability, and anti-tumor activity (Table 1) (Clinical Trial Identification #: NCT03393936). A Phase I study for Axl-CAR-T cell therapy for patients with lung cancer is also being initiated to evaluate safety, tolerability, and preliminary efficacy (Clinical Trial Identification #: NCT03198052).

### 4.5. Natural Products

#### 4.5.1. Viscum album

Mistletoe extract prepared from *Viscum album* extract (VAE) has demonstrated anti-Axl activities (Table 1). VAE treatment decreased Axl expression and phosphorylation and impaired colony formation and tumor cell proliferation of naïve cells as well as cisplatin- and erlotinib-resistant cells [152].

#### 4.5.2. Celastrol

Celastrol is a pentacyclic triterpenoid extracted from *Tripterygium wilfordii* Hook F and *Celastrus regelii*, L., which has anti-Axl activities in non-small-cell lung cancer cells (Table 1). Celastrol reduces Axl expression and impairs tumor cell migration and proliferation in both naïve and gefitinib-resistant non-small-cell lung cancer cells [153].

#### 4.5.3. Yuanhuadine

Yuanhuadine is a daphnane diterpenoid from the flowers of *Daphne genkwa*, which has previously shown anti-proliferative effects in human lung cancer cells (Table 1) [154]. Yuanhuadine decreases Axl expression in both naïve and gefitinib- and osimertinib-resistant non-small-cell lung cancer cells [155]. Yuanhuadine suppresses tumor growth in vivo [155] and synergistically inhibits the growth of EGFR-TKI-resistant cells in vitro and in vivo [156].

## 5. Conclusions

Receptor tyrosine kinases have served as pharmacological targets in cancer treatment due to their frequent dysregulations, including gene amplifications, overexpression, and activating mutations, which promote tumor development, progression, and metastasis. The Gas6/Axl signaling pathway has gained significant attention as a therapeutic target due to its implications in cancer progression, metastasis, and therapeutic resistance [19,20,21]. In addition, studies demonstrate that the Gas6/Axl signaling pathway also modulates the tumor microenvironment [22]. Gas6 is secreted by both cancer and stromal cells [76,157,158,159,160,161,162,163], and studies demonstrate that Axl is also expressed by cancer and stromal cells [22]. Therefore, targeting the Gas6/Axl pathway offers an attractive and promising approach to impair tumor progression and dissemination.

In addition, Axl overexpression is associated with resistance to conventional anticancer treatments, such as chemotherapy and radiotherapy, as well as targeted therapies [60,67,77,109,110,115,126,155,164,165,166,167,168,169,170]. Studies demonstrate that the Axl targeting of resistant tumors can restore sensitivity. Hence, combination therapy with Axl inhibitors offers a rational treatment strategy to overcome therapeutic resistant tumors. Furthermore, Axl inhibition has been shown to modulate the tumor immune microenvironment by multiple mechanisms [22]. Chimeric antigen receptor T cells against Axl have been developed and studied preclinically [149,150,151], and a number of clinical studies are ongoing to evaluate the combination therapy of small molecule Axl inhibitors with immune checkpoint inhibitors. Since Axl is involved in multiple facets of the hallmarks of cancer, results from the ongoing clinical trials will highlight its implications in therapeutic resistance, immune evasion, and metastasis while also demonstrating its safety, tolerability, and efficacy.

Overall, a number of strategies have been employed to target Gas6 and Axl, including small molecule inhibitors, soluble receptors, monoclonal antibodies, CAR-T cell therapy, and natural compounds. These inhibitors have been tested as a monotherapy as well as combination therapies. While targeting the Gas6/Axl pathway has shown promising preclinical and clinical efficacy, targeting Gas6 or Axl as a monotherapy raises a concern about a possible narrow therapeutic index due to its expression and regulation of tumor growth in various cell types of the tumor. The Gas6/Axl pathway has been implicated in drug resistance and immune evasion, and preclinical and clinical studies have demonstrated that combination therapy may exhibit synergistic antitumor activity and drug selectivity. Hence, rational combination approaches and the selection of an appropriate patient population remain a necessity.

Although Axl overexpression is associated with poor clinical prognosis and outcome in multiple cancer types [20], the histological confirmation of Axl expression or activation is often absent in clinical study inclusion criteria. Furthermore, as Gas6 mRNA expression may not predict breast cancer outcomes [171], the assessment of Gas6 protein expression or serum levels, as a potential biomarker for patient response and outcome, is also warranted. Since small molecule tyrosine kinase inhibitors often have multiple targets, results from clinical trials utilizing a monoclonal antibody against Axl (i.e., Axl-107-MMAE and BA301), a chimeric antigen receptor T (CAR-T) cell against Axl (CCT301 CAR-T), and a soluble receptor against Gas6 (ABV-500) are highly anticipated and will offer efficacy and toxicity profiles and guidance for the future development of treatment strategies. Still, preclinical and clinical findings for various Gas6 and Axl inhibitors are favorable and make targeting this axis an attractive and promising approach to impair tumor progression and dissemination.

## Figures and Tables

**Table 1:** Gas6 and Select Class of Axl Inhibitors.

Inhibitor/Developer	Target	Type	Indications	Phase	Strategy	Status
Warfarin	Gas6	Vitamin K agonist	Pancreatic cancer, lung cancer, melanoma, breast cancer	Preclinical/ Phase I	Monotherapy	Withdrawn
MYD1/MYD1-72 Stanford University	Gas6	Soluble receptor	Ovarian cancer, pancreatic cancer	Preclinical	Monotherapy/ Combination	-
AVB-500 Aravive/Stanford University	Gas6	Soluble receptor	Ovarian, renal cell carcinoma, pancreatic adenocarcinoma	Phase I/II	Monotherapy/ Combination	Active, not recruiting/ Recruiting
20G7-D9 Oribase Pharma/French Research Agency of Health/University of Montpellier/Regional Clinical Cancer Center	Axl	Monoclonal antibody	Pancreatic cancer, breast cancer	Preclinical	Monotherapy	-
Axl-107-MMAE Genmab/Oncode Institute, The Netherlands Cancer Institute	Axl	Antibody drug conjugate	Cervical cancer, lung cancer, melanoma	Phase I/II	Monotherapy	Active, not recruiting
BA3011 BioAtla	Axl	Antibody drug conjugate	Lung cancer, prostate cancer, ovarian cancer, pancreatic cancer	Phase I/II	Monotherapy/ Combination	Not yet recruiting/ Recruiting
YW327.6S2 Genentech	Axl	Monoclonal antibody	NSCLC and breast cancer	Preclinical	Combination	-
Mab173 University of Southern California	Axl	Monoclonal antibody	Kaposi sarcoma	Preclinical	Monotherapy	-
Axl-CAR-T Beijing Institute of Radiation Medicine	Axl	CAR-T cells	Triple-negative breast cancer	Preclinical	Monotherapy	-
Axl-CAR.C7R XinJiang Medical University	Axl	CAR-T cells	Triple-negative breast cancer	Preclinical	Monotherapy	-
CCT301 CAR-T PerHum Therapeutics	Axl	CAR-T cells	Renal cell carcinoma	Phase I/II	Monotherapy	Active, not recruiting
Axl-CAR-T Hunan Zhaotai Yongren Medical Innovation/Guangdong Zhaotai InVivo Biomedicine	Axl	CAR-T cells	Lung cancer	Phase I	Monotherapy	Recruiting
Viscum album	Axl	Natural product	NSCLC	Preclinical	Monotherapy	-
Celastrol	Axl	Natural product	NSCLC	Preclinical	Combination	-
Yuanhuadine	Axl	Natural product	NSCLC	Preclinical	Combination	-

**Table 2:** Type I kinase inhibitors against Axl.

Inhibitor/Developer	Target(s)	IC50 for Axl	Indications Approved by the FDA				
Crizotinib (PF-02341066) Pfizer	Axl, **Alk,** **c-Met**, Ron	in vitro: 294 nM	Approved for patients with ALK- or ROS1-positive NSCLC and pediatric ALK-positive anaplastic large-cell lymphoma				
Bosutinib (SKI-606) Pfizer	Axl, **Src kinases**, **Abl**, TGF, BMP	in vitro: 0.56 μM cells: 1.65 μM	Approved for patients with chronic, accelerated, or blast phase Philadelphia chromosome chronic myelogenous leukemia				
Sunitinib (SU11248) Pfizer	Axl, Kit, Flt3, **PDGFR**, **VEGFR2**	in vitro: 9 nM	Approved for renal cell carcinoma and imatinib-resistant gastrointestinal stromal tumor and metastatic pancreatic neuroendocrine tumors				
**Inhibitor/Developer**	**Target(s)**	**IC50 for Axl**	**Clinical Trial #**	**Phase**	**Strategy**	**Status**	**Indications**
Bemcentinib (BGB324, R428) Rigel Pharmaceuticals/BerGen BIO	Axl	in vitro: 14 nM cells: <30 nM	NCT03965494	I	Monotherapy	Recruiting	Glioblastoma
			NCT02922777	I	Combination	Recruiting	NSCLC
			NCT03649321	I/II	Combination	Recruiting	Pancreatic adenocarcinoma
			NCT02872259	I/II	Combination	Recruiting	Melanoma
			NCT03824080	II	Monotherapy	Active, not recruiting	Acute myeloid leukemia (AML), Myelodysplastic syndrome
			NCT03654833	II	Combination	Recruiting	Mesothelioma
			NCT03184558	II	Combination	Completed	TNBC, TN-inflammatory breast cancer
			NCT03184571	II	Combination	Recruiting	NSCLC
Amuvatinib (MP-470) Astex Pharmaceuticals	**c-Kit**, Axl, c-Met, **PDGFRα**, FLT3, c-Ret, RAD51	cells: <1 μM	NCT00881166	I	Combination	Completed	Invasive malignancy except non-melanoma skin cancers or cervical carcinoma in situ
			NCT00894894	I	Monotherapy	Completed	Unresectable or metastatic solid tumor
			NCT01357395	II	Monotherapy	Completed	Small-cell lung cancer (SCLC)
Dubermatinib (TP-0903) Tolero Pharmaceuticals	**Axl**, Aurora A and B, JAK2, Alk, Abl, Mer	in vitro: 27 nM cells: 222 nM	NCT02729298	I	Monotherapy	Active, not recruiting	Advanced metastatic or progressive solid tumor (Phase 1b: EGFR positive NSCLC; BRAF-, KRAS-, or NRAS-mutated colorectal carcinoma; recurrent ovarian carcinoma; BRAF-mutated melanoma)
			NCT03013998	I/II	Monotherapy/ Combination	Recruiting	AML
			NCT04518345	I/II	Monotherapy	Recruiting	AML
			NCT03572634	I/II	Combination	Terminated	Chronic lymphocytic leukemia, small lymphocytic lymphoma
S49076 Servier	**Met mutants**, **Axl**, Mer, **FGFRs**	in vitro: 7 nM cells: 56 nM	ISRCTN00759419	I	Monotherapy	Completed	Advanced solid tumors
			ISRCTN11619481	I/II	Combination	Completed	GBM
SNS-314 Sunesis Pharmaceuticals	**Aurora-A, -B, -C,** Trk A/B, Flt4, Axl, c-Raf	in vitro: 84 nM	NCT00519662	I	Monotherapy	Completed	Advanced solid tumors
NA80x1 Wyeth-Ayerst Research/ Max Planck Institute of Biochemistry	Axl	in vitro: 12.8 μM cells: 4.11 μM	Preclinical	-	Monotherapy	-	-
SGI-7079 Northwestern University	**Axl**, Met, Mer, Yes, Ret, Flt3	in vitro: 58 nM cells: <1 μM	Preclinical	-	Monotherapy/ Combination	-	-
DP-3975 Deciphera Pharamaceuticals	Axl	cells: 0.1 μM	Preclinical	-	Monotherapy	-	-
UNC569 University of North Carolina	**Mer**, Tyro3, Axl	in vitro: 37 nM	Preclinical	-	Monotherapy	-	-
UNC2025 University of North Carolina	**Mer**, **Flt3**, Axl, Tyro3	in vitro: 1.6 nM	Preclinical	-	Monotherapy	-	-

**Bold**: preferred target.

**Table 3:** Type II kinase inhibitors against Axl.

Inhibitor/ Developer	Target(s)	IC50 for Axl	Indications Approved by the FDA				
Cabozantinib (BMS-907351) Exelixis	**VEGFR2**, c-Met, Ret, Tie2, c-Kit, Axl	in vitro: 7 nM cells: 42 nM	Approved for patients with advanced renal cell carcinoma, sorafenib-resistant hepatocellular carcinoma, and combination therapy with nivolumab for advanced renal cell carcinoma				
**Inhibitor/** **Developer**	**Target(s)**	**IC50 for Axl**	**Clinical Trial #**	**Phase**	**Strategy**	**Status**	**Indications**
Foretinib (GSK1363089) GlaxoSmithKline	**c-Met**, **VEGFR2**, Ron, Axl	in vitro: 11 nM cells: <100 nM	NCT01138384	I/II	Combination	Completed	Invasive, HER2-positive breast cancer
			NCT01147484	II	Monotherapy	Completed	Recurrent triple-negative breast cancer
			NCT00726323	II	Monotherapy	Completed	Renal cell carcinoma
			NCT00725712	II	Monotherapy	Completed	Metastatic gastric carcinoma
Sitravatinib (MGCD516) Mirati Therapeutics	**c-Kit**, PDGFRα/β, Axl, c-Met	in vitro: 1.5 nM cells: 250–800 nM	NCT04123704	II	Monotherapy	Recruiting	Metastatic TNBC
			NCT03680521	II	Combination	Active, not recruiting	ccRCC
Glesatinib (MGCD265) Mirati Therapeutics	**c-Met**, RON, Axl, VEGFR	Ν/A	NCT02954991	II	Combination	Active, not recruiting	NSCLC
Rebastinib (DCC-2036) Deciphera Pharmaceuticals	**Bcr-Abl**, Axl, Flt3, VEGFR2, Src	in vitro: 42 nM	NCT02824575	I	Combination	Recruiting	HER2-negative breast cancer
			NCT03717415	I/II	Combination	Recruiting	Advanced or metastatic solid tumor
			NCT03601897	I/II	Combination	Recruiting	Locally advanced or metastatic solid tumor
			NCT00827138	I	Monotherapy	Completed	CML
Merestinib (LY2801653) Eli Lilly and Company	**c-Met**, MST1R, Axl, ROS1, Flt3	in vitro: 11 nM cells: 2 nM	NCT02711553	II	Combination	Active, not recruiting	Advanced or metastatic biliary tract cancer
			NCT02920996	II	Monotherapy	Active, not recruiting	NSCLC
			NCT03125239	I	Combination	Completed	Relapsed or refractory AML
BMS-777607 Aslan Pharmaceuticals	Axl, Ron, **c-Met**, Tyro3	in vitro: 1.1 nM	NCT01721148	I	Monotherapy	Completed	Advanced or metastatic solid tumor
			NCT00605618	I/II	Monotherapy	Completed	Advanced or metastatic solid tumor
RXDX-106 (CEP-40783) Ignyta, Inc.	**Axl**, Tyro3, Mer, c-Met	in vitro: 7 nM	NCT03454243	I	Monotherapy	Terminated	Locally advanced or metastatic solid tumor
NPS-1034 Neopharm	**c-Met, Axl**	in vitro: 10 nM	Preclinical	-	Combination	-	-
LDC1267 Lead Discovery Center	**Axl, Tyro3**, **Mer**, c-Met, Src	in vitro: 29 nM	Preclinical	-	Monotherapy/ Combination	-	-

**Bold**: preferred target.
